# Supplementary material for: NAA60 facilitates LRRC8A- and LRRC8D-mediated platinum drug uptake
Source: Commun Biol. 2025 Oct 6;8:1431. doi: 10.1038/s42003-025-08826-x (PMC12501270; doi:10.1038/s42003-025-08826-x)
Supplement: Supplementary file 2 — Supplementary Information [file 42003_2025_8826_MOESM2_ESM.pdf]

Supplementary Information for

NAA60 facilitates LRRC8A- and LRRC8D-mediated platinum drug uptake

Carmen Alexandra Widmer[1,2], Anna Moyseos[1,2], Ismar Klebic[1,3], Martina Dettwiler[1], Martín González-Fernández[1,2,4], Ewa Gogola[5], Myriam Siffert[1], Natasha Buchs[6], Sophie Braga-Lagache[6], Anne-Christine Uldry[6], Jos Jonkers[5], Manfred Heller[6], Sven Rottenberg[1,2,3,4,5]

Authors' affiliations:

[1]Institute of Animal Pathology, Vetsuisse Faculty, University of Bern, 3012 Bern, Switzerland

[2]Bern Center for Precision Medicine, University of Bern, 3012 Bern, Switzerland

[3]COMPATH, Institute of Animal Pathology, Vetsuisse Faculty, University of Bern, 3012 Bern, Switzerland

[4]Cancer Therapy Resistance Cluster, Department for BioMedical Research (DBMR), University of Bern, 3012 Bern, Switzerland

[5]Division of Molecular Pathology, The Netherlands Cancer Institute, 1066CX Amsterdam, The Netherlands

[6]Proteomics and Mass Spectrometry Core Facility, Department for Biomedical Research (DBMR), University of Bern, 3012 Bern, Switzerland

Supplementary Figures

Supplementary Fig.1 Loss of NAA60 leads to increased levels of Golgi fragmentation in KB1PM5 cells

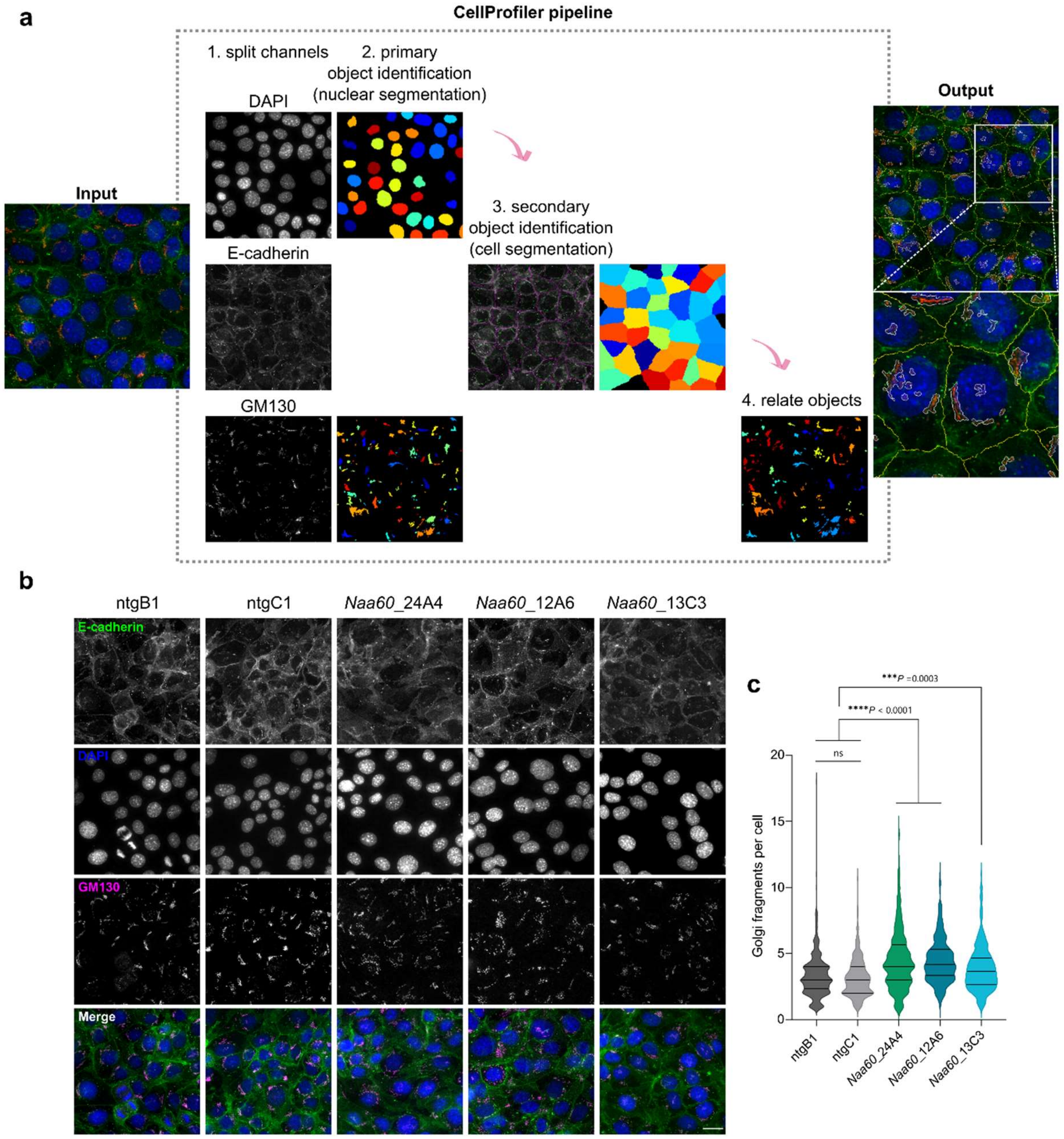

a Layout of the CellProfiler pipeline to quantify Golgi fragments per cell: Input images containing DAPI, E-cadherin, and GM130 immunofluorescence staining were subjected to a pipeline, where first these channels were split to separate image files (1) next, the nuclei and Golgi fragments were identified as primary objects (2). As a next step, the cells were segmented based on the nuclear segmentation and the E-cadherin staining (3). Finally, the Golgi objects could be related back to each individual cell and quantified (4). **b** Representative immunofluorescence staining for the Golgi

fragmentation quantification. The scale bar represents 20µm **c** Quantification of the Golgi fragments per cell using the CellProfiler pipeline. For each cell line and replicate 250-300 nuclei were quantified. Lines at median and quartiles of three independent replicates are shown (ordinary one-way ANOVA followed by Tukey's multiple comparisons test).

Supplementary Fig.2 Reintroduction of *Naa60* cDNA re-sensitizes *Naa60* knockout cell lines towards Pt-based agents cisplatin and carboplatin, as well as blasticidin S

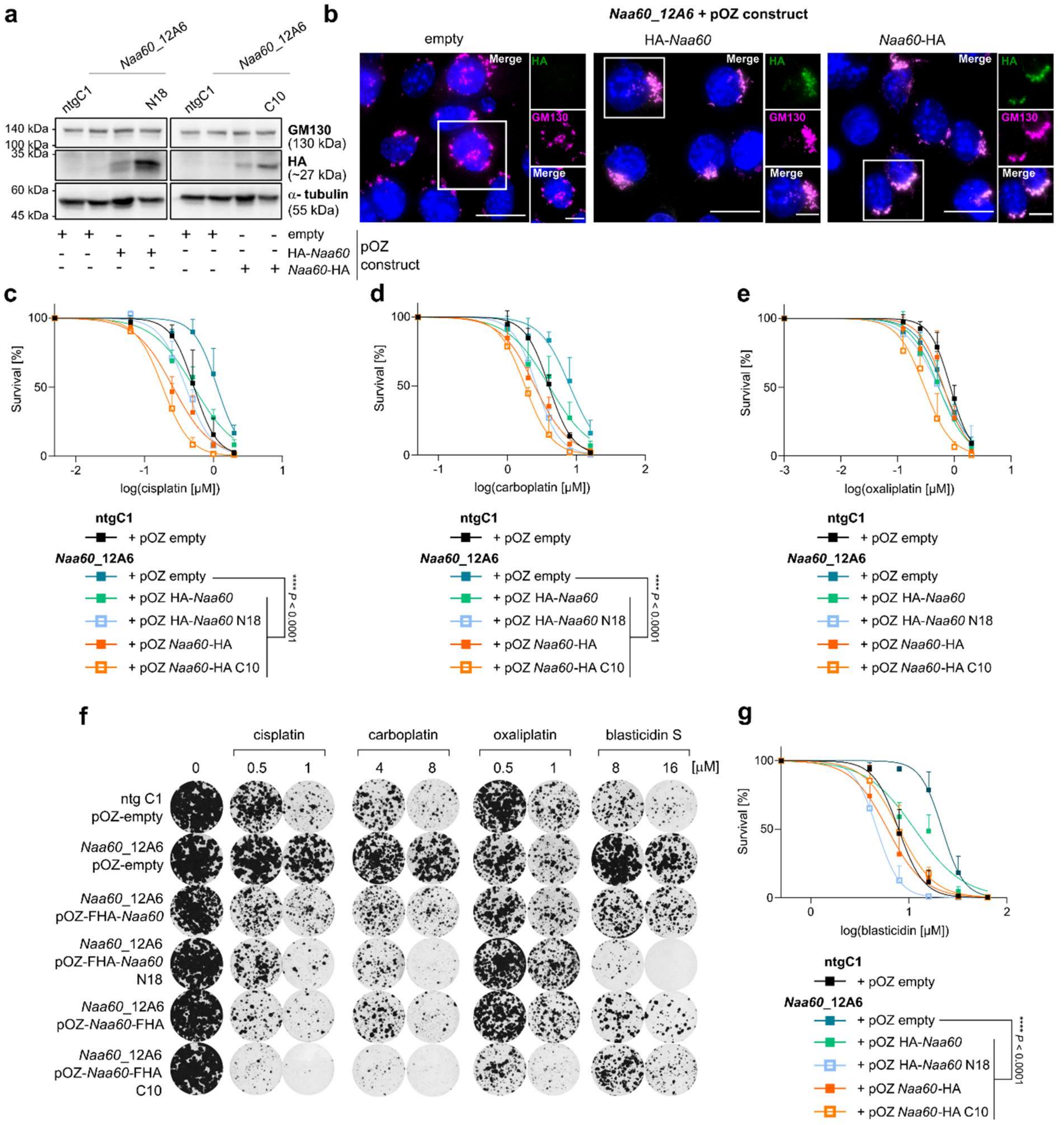

a Western blot expression control of the pOZ HA-*Naa60* or pOZ *Naa60*-HA constructs in the *Naa60* knockout line *Naa60*_12A6. **b** Immunofluorescence imaging of the HA-tagged NAA60 together with a double staining of the Golgi Marker 130 (GM130). The scale bar represents 20µm on the full-size images and 10µm on the cropped image sections **c-g** Clonogenic survival assays of the different rescue cell lines treated with cisplatin, carboplatin, oxaliplatin, and blasticidin S for 24h with the indicated drug concentrations. Representative images of selected lines and concentrations are shown. Data represent mean ± SD of three independent replicates and were fitted to

a non-linear regression dose-response curve (log(inhibitor) vs. normalized response - Variable slope). *P*-values are calculated by one-way ANOVA followed by Tukey's multiple comparisons test for the log(IC50) values of the survival curves.

Supplementary Fig.3 NAA60-deficient organoids are resistant to cisplatin and carboplatin.

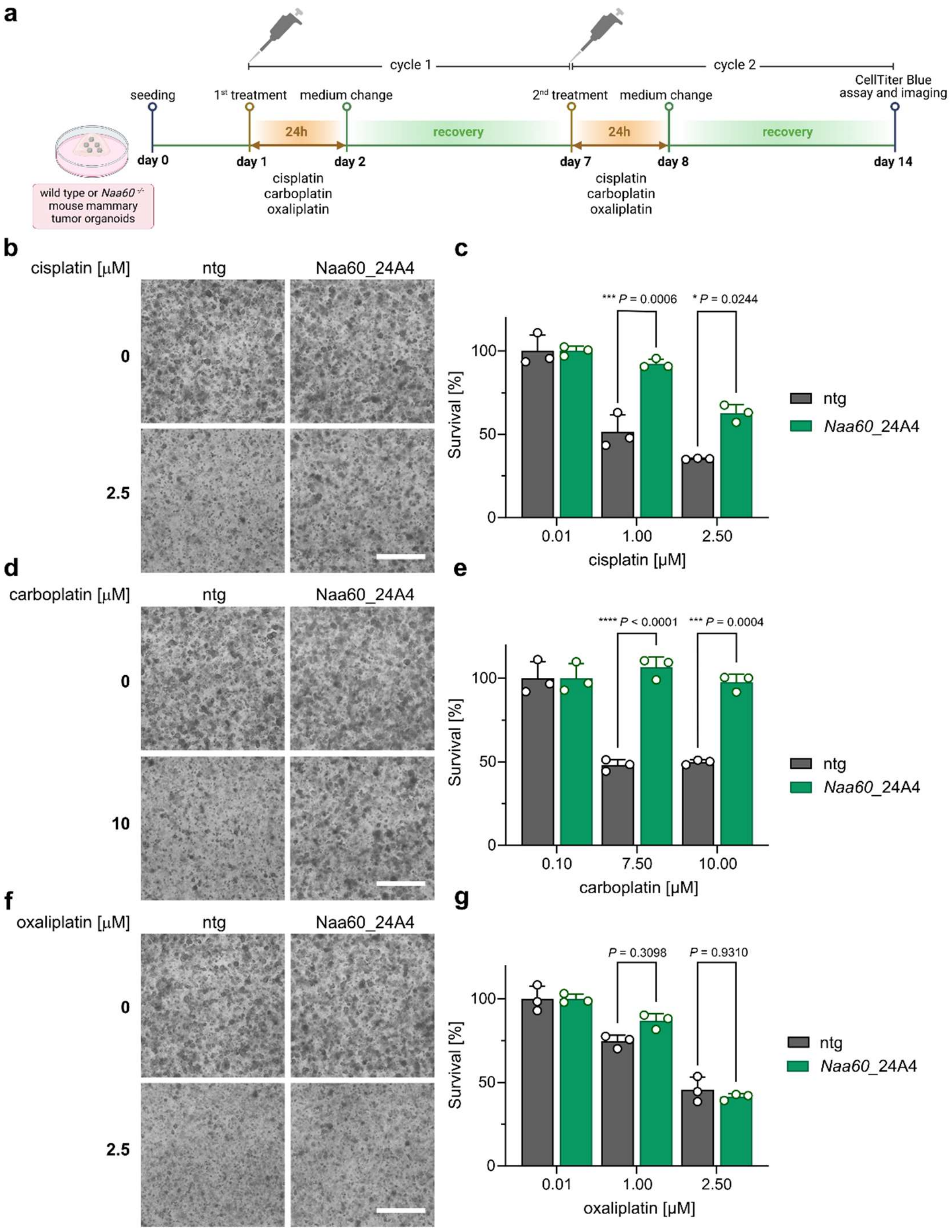

a Outline of the organoids experiment which included two cycles of drug treatment on day 1 and day 7 for 24h each, afterwards the medium was changed to fresh growth medium, and the organoids were left to recover. Imaging and CellTiter Blue viability assay were performed on day 14. Created in BioRender. Widmer, C. (2025) https://BioRender.com/dd5c8lp. **b+d+f** Representative images of the growth assay using a wild-type (ntg) or a *Naa60* knockout (*Naa60*_24A4) organoid line with cisplatin,

carboplatin, or oxaliplatin treatment. The images were taken on day 14 of the assay, right before the CellTiter-Blue® viability assay was performed. The scale bar equals 1mm. **c+e+g** Quantification of cisplatin, carboplatin or oxaliplatin treatment response. Data represents the mean ± SD of 2 biological replicates, which consisted of 3 technical replicates each.

Supplementary Fig.4 Drug panel testing reveals resistance towards blasticidin S upon loss of *Naa60*

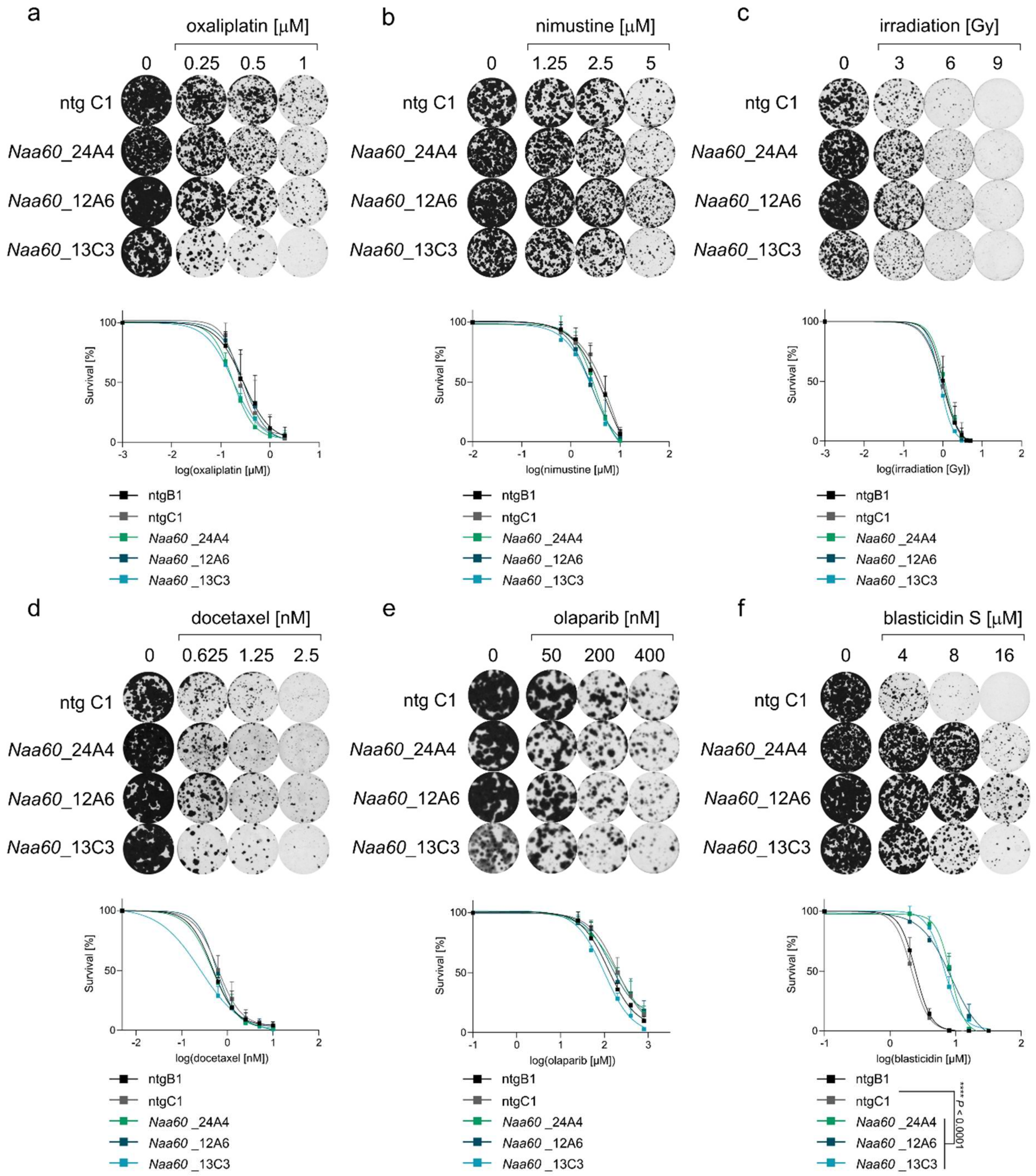

a-f Clonogenic survival assays of wild-type (ntgB1 and ntgC1) and NAA60-deficient (*Naa60*_24A4, *Naa60*_12A6, and *Naa60*_13C3) cell lines treated with oxaliplatin, nimustine, ionizing irradiation, docetaxel, olaparib, and blasticidin S. Representative images of selected lines and concentrations are shown. Drug treatments were carried out over the course of 24h. The doses given for ionizing irradiation refer to a one-time treatment. Data represent mean ± SD (oxaliplatin N=3, nimustine N=4, irradiation N=2,

docetaxel N=3, Olaparib N=4, and blasticidin S N=3) and were fitted to a non-linear regression dose-response curve (log(inhibitor) vs. normalized response -Variable slope). *P*-values are calculated by one-way ANOVA followed by Tukey's multiple comparisons test for the log(IC50) values of the survival curves.

Supplementary Fig.5 LRRC8A and LRRC8D N-termini are a substrate for NAA60

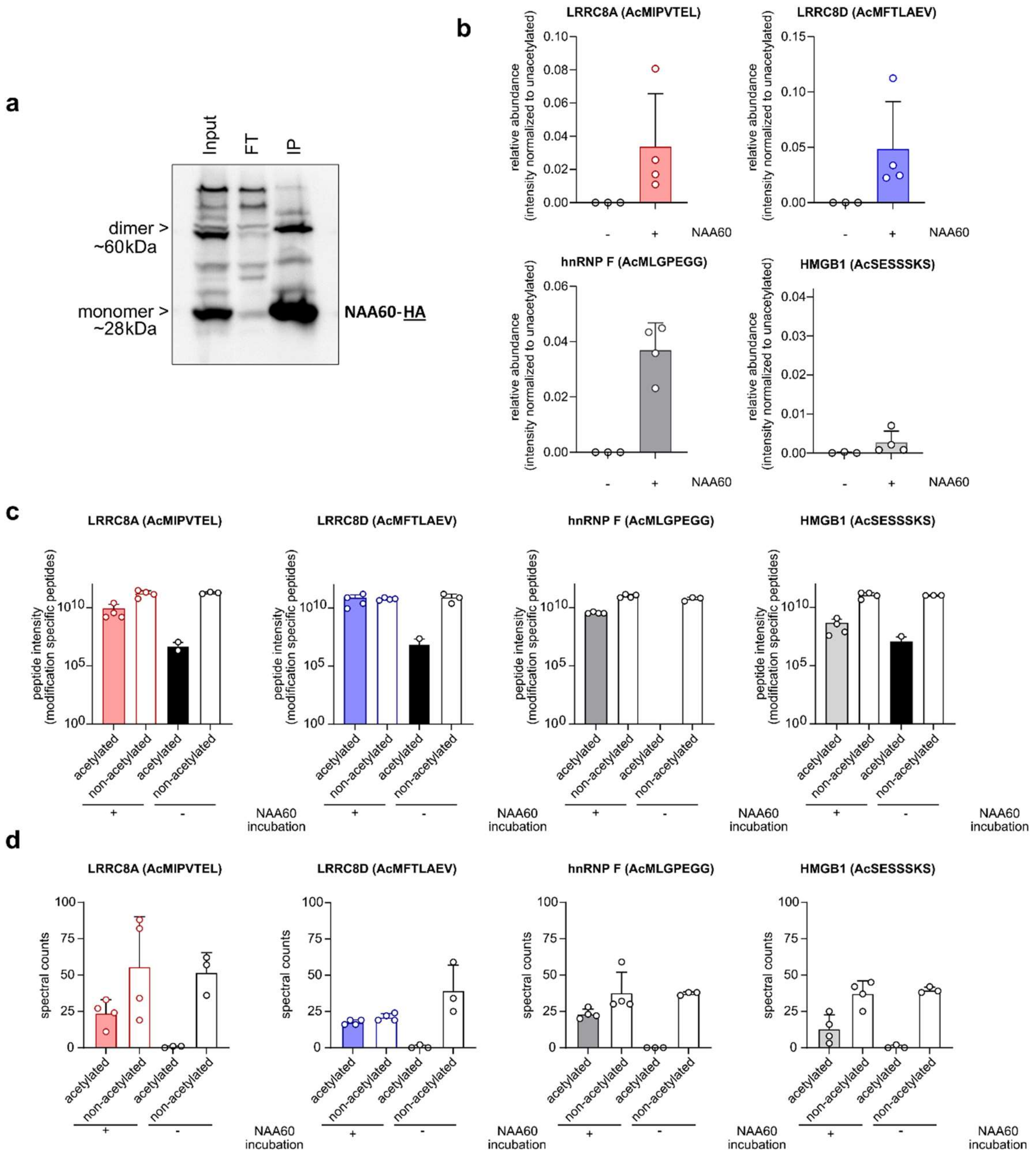

a Western blot of the NAA60-HA immunoprecipitation using anti HA- magnetic beads. The beads enriched with the NAA60 enzyme were subsequently used in the *in vitro* acetylation assay (FT= flow through, IP= immunoprecipitation). **b** *In vitro* acetylation assay of immunoprecipitated NAA60-HA with peptides containing the first seven amino acids of either LRRC8A, LRRC8D, hnRNP F, or HMGB1. N=4 for the enzyme incubated samples, N=3 of the empty bead incubated samples. All replicates were performed with beads from the same enzyme harvest. **c** raw peptide intensity levels and **d** spectral counts.

Supplementary Fig.6 NAA60 and LRRC8A or LRRC8D are in close proximity within the cell

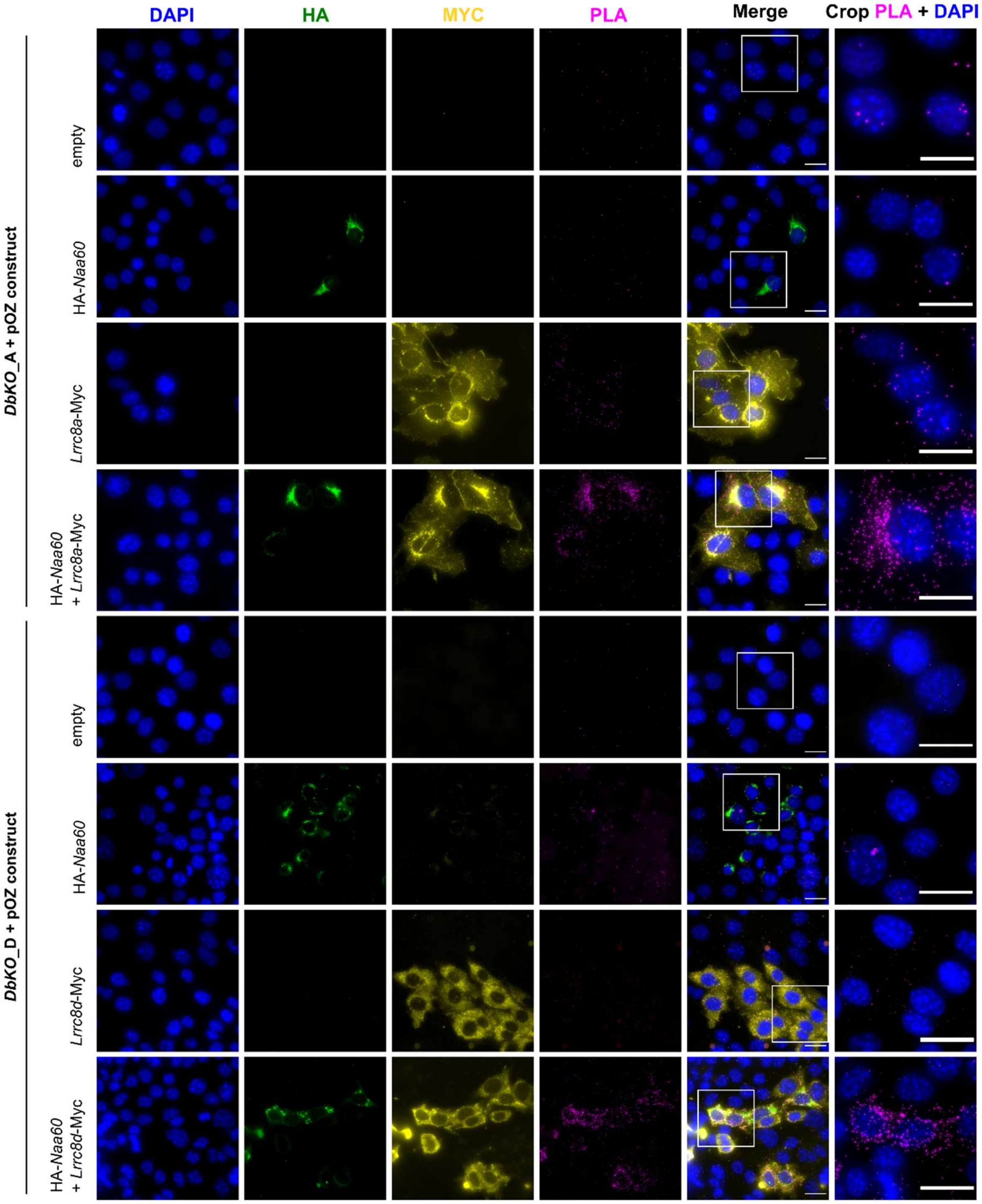

PLA staining of HA-NAA60 and LRRC8A-MYC in the polyclonal *DbKO*_A rescue lines or HA-NAA60 and LRRC8D-MYC in the polyclonal *DbKO*_D rescue line. To visualize the rescue protein expression, a secondary antibody staining against the primary antibodies from the PLA was performed after finishing the reaction of the PLA. Images from one representative replicate are shown. The scale bars of both full size and cropped images equal 20µm.

Supplementary Fig.7 NAA60 and LRRC8D reconstitution into the double knockout cell line *DbKO*_D

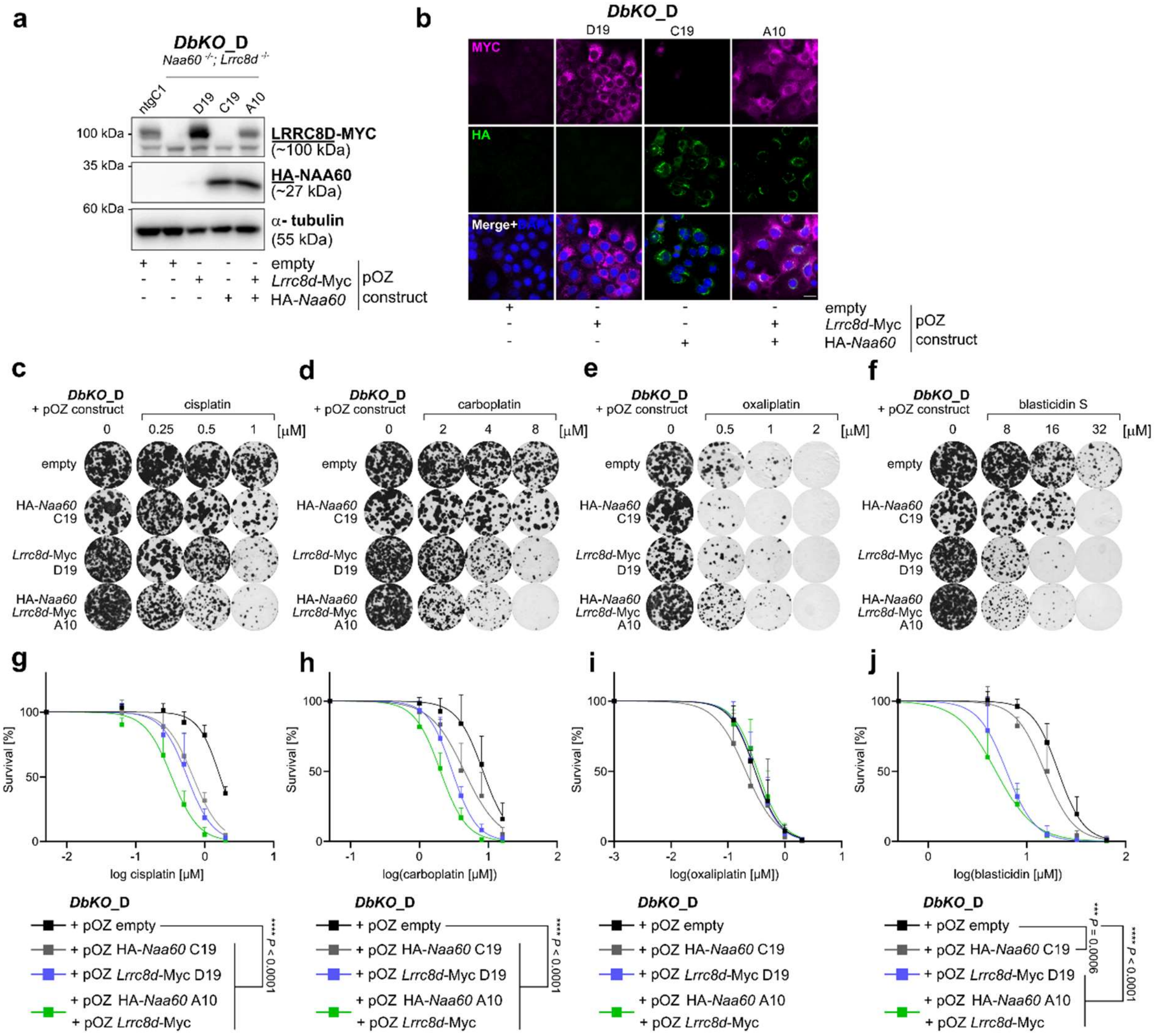

a Western blot rescue control, where either HA-NAA60 or LRRC8D-MYC or both proteins were reintroduced into a double knockout line *DbKO*_D (*Naa60*$^{-/-}$; *Lrrc8d*$^{-/-}$). **b** Representative immunofluorescence images of the HA or MYC tags on the proteins, which were rescued into the *DbKO*_D line. The scale bar equals 10µm **c-f** Clonogenic survival assays of the different monoclonal *DbKO*_D rescue cell lines treated with cisplatin, carboplatin, oxaliplatin, or blasticidin S. Representative images of selected lines and concentrations are shown. **g-j** Quantification of clonogenic growth assays using the *DbKO*_D-based rescue cell lines in the presence of cisplatin, carboplatin, oxaliplatin, or blasticidin S. Data represent mean ± SD of four independent replicates and were fitted to a non-linear regression dose-response curve (log(inhibitor) vs. normalized response -Variable slope). *P*-values are calculated by one-way ANOVA followed by Tukey's multiple comparisons test for the log(IC50) values of the survival curves.

Supplementary Fig.8 Source data uncropped Western Blots for main figures

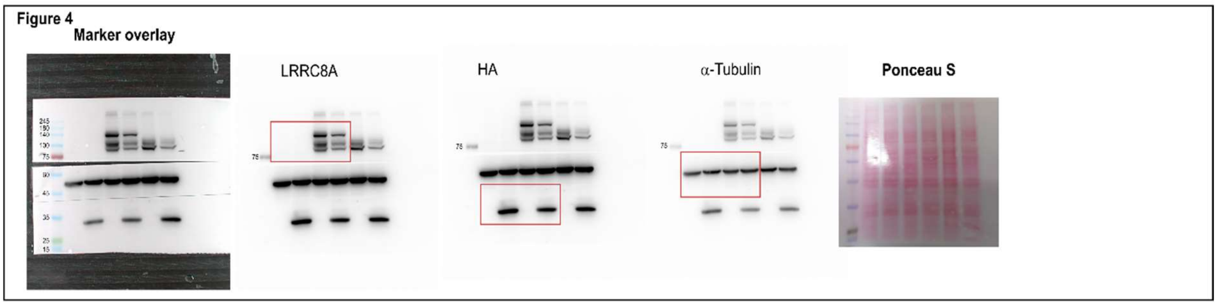

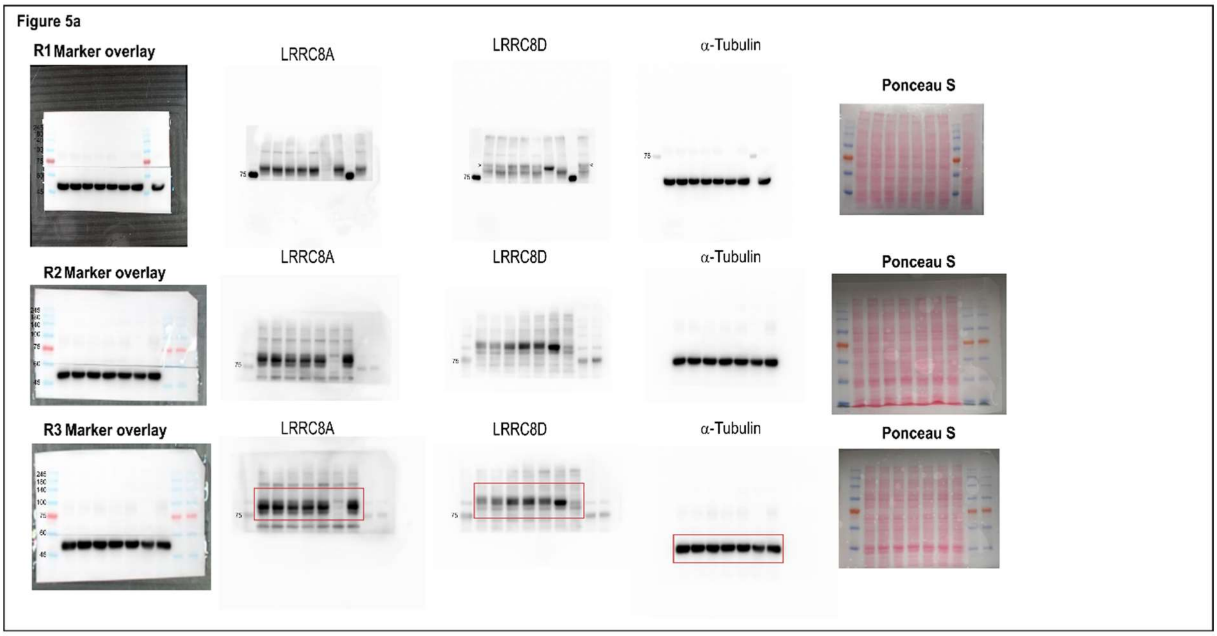

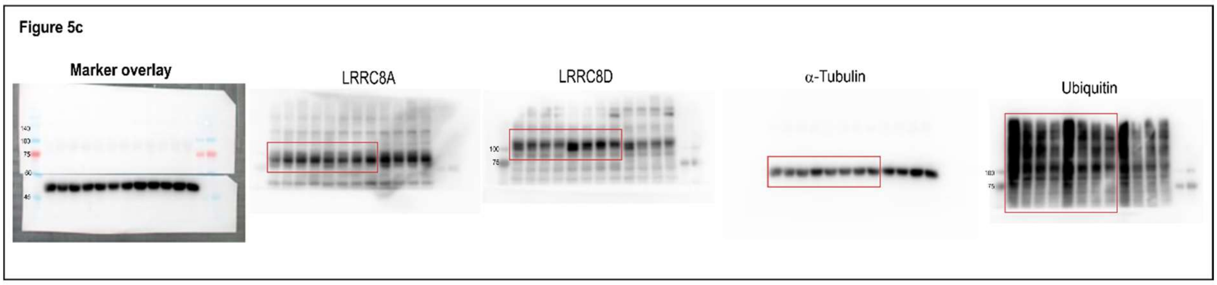

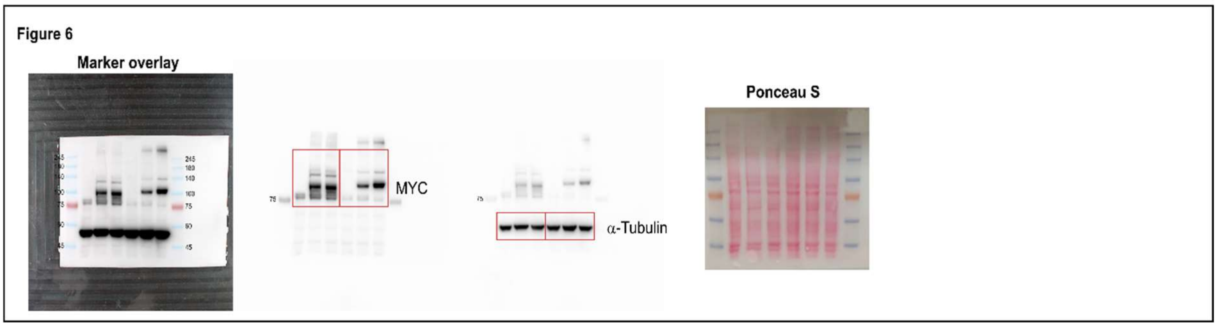

Supplementary Fig.9 Source data uncropped Western Blots for supplementary figures.

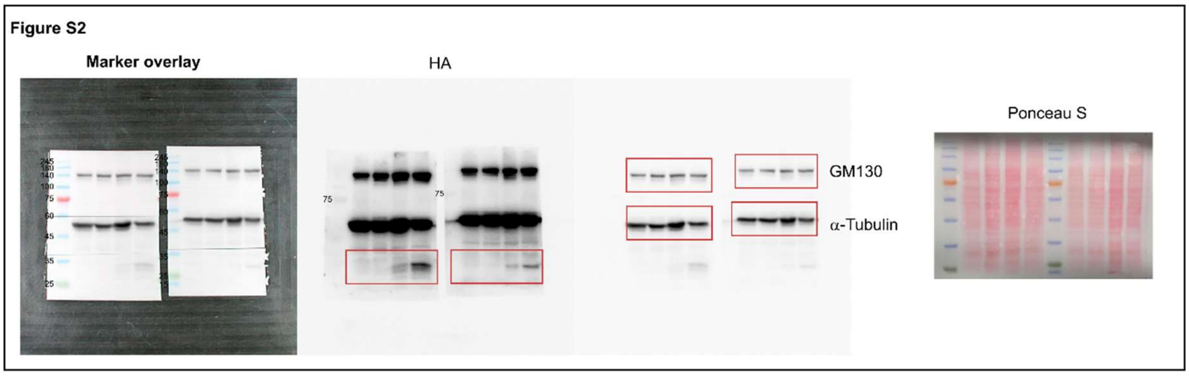

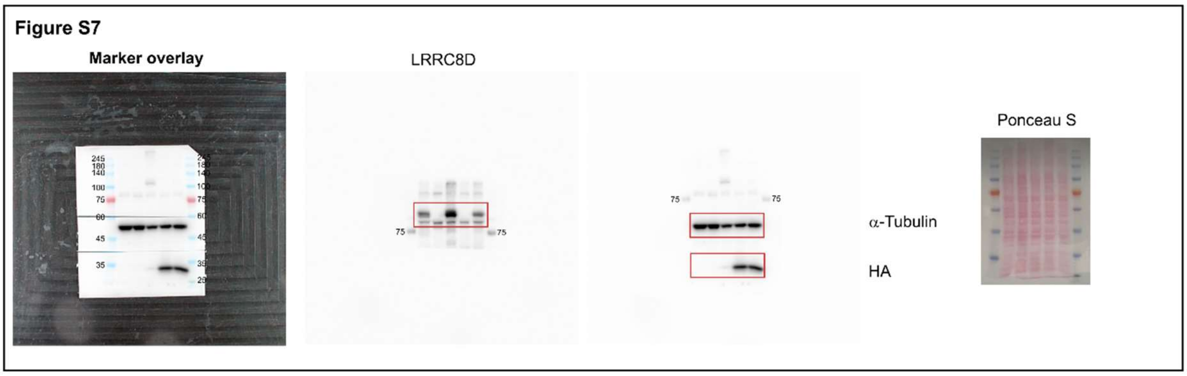

Supplementary Table 1. Naa60-targeting gRNA sequences

Oligo name	Sequence 5'-3'	gRNA name
gRNA-ntg-ms-f	CACCGTGATTGGGGGTCGTTCGCCA	ntg
gRNA-ntg-ms-r	AAACTGGCGAACGACCCCCAATCAC	
gRNA-Naa60-1f	CACCGAGTTGCATACATCCTCAGTC	1
gRNA-Naa60-1r	AAACGACTGAGGATGTATGCAACTC	
gRNA-Naa60-2f	CACCGTCATATTTCAACCACCGCCC	2
gRNA-Naa60-2r	AAACGGGCGGTGGTTGAAATATGAC	
gRNA-Naa60-3f	CACCGCCTCGAATGGAGTAGTAGTA	3
gRNA-Naa60-3r	AAACTACTACTACTCCATTCGAGGC	
gRNA-Naa60-4f	CACCGCTACTACTACTCCATTCGAG	4
gRNA-Naa60-4r	AAACCTCGAATGGAGTAGTAGTAGC	

Monoclonal knockout cell lines:	gRNAs used for knockout generation:
Naa60 _**24**A4	2+4
Naa60 _**12**A6	1+2
Naa60 _**13**C3	1+3
