# Supplementary material for: NAA60 facilitates LRRC8A- and LRRC8D-mediated platinum drug uptake
Source: Commun Biol. 2025 Oct 6;8:1431. doi: 10.1038/s42003-025-08826-x (PMC12501270; doi:10.1038/s42003-025-08826-x)
Supplement: Supplementary file 3 — Description of Additional Supplementary Files [file 42003_2025_8826_MOESM3_ESM.pdf]

Description of Additional Supplementary Files

File name: Supplementary Data 1

Description: Results of the genome-wide CRISPR/Cas9 screen with cisplatin treatment

File name: Supplementary Data 2

Description: The source data behind the graphs of the main figures

File name: Supplementary Data 3

Description: The source data behind the graphs of the supplementary figures
