# Supplementary material for: NAA60 facilitates LRRC8A- and LRRC8D-mediated platinum drug uptake
Source: Commun Biol. 2025 Oct 6;8:1431. doi: 10.1038/s42003-025-08826-x (PMC12501270; doi:10.1038/s42003-025-08826-x)
Supplement: Supplementary file 7 — reporting summary [file 42003_2025_8826_MOESM7_ESM.pdf]

nature portfolio

Corresponding author(s): Sven Rottenberg

Last updated by author(s): Aug 6, 2025

Reporting Summary

Nature Portfolio wishes to improve the reproducibility of the work that we publish. This form provides structure for consistency and transparency in reporting. For further information on Nature Portfolio policies, see our Editorial Policies and the Editorial Policy Checklist.

Statistics

For all statistical analyses, confirm that the following items are present in the figure legend, table legend, main text, or Methods section.

n/a	Confirmed	
☐	☒	The exact sample size (*n*) for each experimental group/condition, given as a discrete number and unit of measurement
☐	☒	A statement on whether measurements were taken from distinct samples or whether the same sample was measured repeatedly
☐	☒	The statistical test(s) used AND whether they are one- or two-sided<br>*Only common tests should be described solely by name; describe more complex techniques in the Methods section.*
☐	☒	A description of all covariates tested
☐	☒	A description of any assumptions or corrections, such as tests of normality and adjustment for multiple comparisons
☐	☒	A full description of the statistical parameters including central tendency (e.g. means) or other basic estimates (e.g. regression coefficient) AND variation (e.g. standard deviation) or associated estimates of uncertainty (e.g. confidence intervals)
☐	☒	For null hypothesis testing, the test statistic (e.g. *F*, *t*, *r*) with confidence intervals, effect sizes, degrees of freedom and *P* value noted<br>*Give P values as exact values whenever suitable.*
☒	☐	For Bayesian analysis, information on the choice of priors and Markov chain Monte Carlo settings
☒	☐	For hierarchical and complex designs, identification of the appropriate level for tests and full reporting of outcomes
☒	☐	Estimates of effect sizes (e.g. Cohen's *d*, Pearson's *r*), indicating how they were calculated

Our web collection on statistics for biologists contains articles on many of the points above.

Software and code

Policy information about availability of computer code

Data collection	SoftWoRx DeltaVision software, FUSION FX7 imaging system (Vilber GmbH), For the CRISPR screen analysis, FASTA files were subjected to quality control with FASTQC and counted with the MaGeCK count function.
Data analysis	GraphPad Prism v9, MAGeCK software, ColonyArea plugin ImageJ version 1.53i, Plot Profile plugin of ImageJ version 2.14.0, fragpipe version 20.0, MaxQuant Version 1.6.14.0, FIJI image processing package of ImageJ 1.8.0, Screen count tables were analyzed on UBELIX (http://ww.id.unibe.ch/hpc), the HPC cluster at the University of Bern, using the publicly available MaGeCK module (Li et al. 2015). Results were processed in R open software (version 4.1.3) using MaGeCKFlute package (Wang et al. 2019), For the analysis of the Helios CyTOF data the premessa R package released by the Parker Institute for Cancer Immunotherapy (https://github.com/ParkerICI/premessa) was used.

For manuscripts utilizing custom algorithms or software that are central to the research but not yet described in published literature, software must be made available to editors and reviewers. We strongly encourage code deposition in a community repository (e.g. GitHub). See the Nature Portfolio guidelines for submitting code & software for further information.

Data

Policy information about availability of data

All manuscripts must include a data availability statement. This statement should provide the following information, where applicable:

- Accession codes, unique identifiers, or web links for publicly available datasets
- A description of any restrictions on data availability
- For clinical datasets or third party data, please ensure that the statement adheres to our policy

All data will be shared upon request by the lead contact with no restrictions. Source data are provided with this paper. Raw sequences of the CRISPR/Cas9 screen with cisplatin are available in European Nucleotide Archive (ENA) under the accession number PRJEB75036 (https://www.ebi.ac.uk/ena/browser/view/PRJEB75036). The plasma membrane proteomics data is deposited in the ProteomeXchange Consortium via the PRIDE partner repository with the dataset identifier PXD035143 and will be made available upon request. The remaining data are available within the Article, Supplementary Information, or Source Data.

Research involving human participants, their data, or biological material

Policy information about studies with human participants or human data. See also policy information about sex, gender (identity/presentation), and sexual orientation and race, ethnicity and racism.

Reporting on sex and gender	No data of human participants was used in this study.
Reporting on race, ethnicity, or other socially relevant groupings	No data of human participants was used in this study.
Population characteristics	No data of human participants was used in this study.
Recruitment	No data of human participants was used in this study.
Ethics oversight	No data of human participants was used in this study.

Note that full information on the approval of the study protocol must also be provided in the manuscript.

Field-specific reporting

Please select the one below that is the best fit for your research. If you are not sure, read the appropriate sections before making your selection.

☒ Life sciences ☐ Behavioural & social sciences ☐ Ecological, evolutionary & environmental sciences

For a reference copy of the document with all sections, see nature.com/documents/nr-reporting-summary-flat.pdf

Life sciences study design

All studies must disclose on these points even when the disclosure is negative.

Sample size	All experiments represented in this manuscript are performed as biological replicates, with at least two or three independent replicates as stated in each figure legend. For the in vivo transplantation studies, the number of mice for each experiment was calculated based on power analysis performed in previous publications from the lab. The number of biological replicates and the number of individual measurements were considered to have enough power to reveal relevant biological insights with sufficient statistical significance.
Data exclusions	No data exclusions were performed.
Replication	All experiments were repeated in multiple independent experiments to ensure the reproducibility of the observed results. Description of the exact number of independent repetitions and technical replicates are indicated in each corresponding figure legend. All attempts were successful.
Randomization	After tumor outgrowth, the mice for the in vivo validation experiments were randomly allocated into the vehicle, cisplatin or carboplatin treatment groups.
Blinding	For the animal experiments, animal technicians, who were blinded regarding the hypothesis, performed the tumor size measurements and treatments. All other experiments used objective quantitative methods and researchers did not have expectations regarding the outcome.

Reporting for specific materials, systems and methods

We require information from authors about some types of materials, experimental systems and methods used in many studies. Here, indicate whether each material, system or method listed is relevant to your study. If you are not sure if a list item applies to your research, read the appropriate section before selecting a response.

Materials & experimental systems

n/a	Involved in the study	
☐	☒	Antibodies
☐	☒	Eukaryotic cell lines
☒	☐	Palaeontology and archaeology
☐	☒	Animals and other organisms
☒	☐	Clinical data
☒	☐	Dual use research of concern
☒	☐	Plants

Methods

n/a	Involved in the study	
☒	☐	ChIP-seq
☒	☐	Flow cytometry
☒	☐	MRI-based neuroimaging

Antibodies

Antibodies used

Primary antibodies:
anti-LRRC8A rabbit polyclonal (Bethyl Laboratories Cat#A304-175A)
anti-LRRC8D rabbit polyclonal (Proteintech Cat#11537-1-AP)
anti-HA.11 Epitope Tag Antibody Clone 16B12 mouse monoclonal (BioLegend Cat#901501)
anti-HA rabbit polyclonal C29F4 (Cell signaling Cat#3724S)
anti-Myc-tag 71D10 rabbit polyclonal (Cell signaling Cat#2278)
anti- Myc-Tag 9B11 mouse monoclonal (Cell signaling Cat#2276)
anti-beta actin mouse monoclonal (Sigma Cat#A1978)
anti-alpha tubulin, mouse monoclonal (Sigma Cat#T5168)
anti-phospho-Histone H2A.X ser139 clone JBW301 (Merck Millipore Cat#05-636)
anti-GM130 Alexa Fluor 488 conjugated (BD Biosciences Cat#560257)
anti-E-cadherin FITC conjugated (BD Biosciences Cat#612130)
anti-ubiquitin rabbit monoclonal (Thermo Scientific Cat#MA5-37950)

Secondary antibodies:
(HRP)-linked anti-mouse IgG (Cell Signaling Cat#7076)
(HRP)-linked anti-rabbit IgG (Cell Signaling Cat#7074)
Goat anti-Mouse IgG (H+L) Cross-Adsorbed Secondary Antibody Alexa Fluor 488 (Thermo Scientific Cat#A11029)
Goat anti-rabbit IgG (H+L) Cross-Adsorbed Secondary Antibody Texas Red (Thermo Scientific Cat#T-2767)
Goat anti-Rabbit IgG (H+L) Highly Cross-Adsorbed Secondary Antibody, Alexa Fluor Plus 555, (Thermo Scientific Cat#A32732)

Validation

All antibodies used in this sutdy were commercially available by vendors and have been validated by the manufacturers. LRRC8A and LRRC8D-targeting antibodiy specificity was additionally confirmed by the use of isogenic knockout control cell lines.

Eukaryotic cell lines

Policy information about cell lines and Sex and Gender in Research

Cell line source(s)

The original mouse cell lines used in this study have been previously described (Evers et al. 2008; Jaspers et al. 2013).

Authentication

All mouse cell lines were authenticated by Brca1/2-specific PCR-based genotyping (mouse) (Evers et al. 2008; Jaspers et al. 2013).

Mycoplasma contamination

Cells have been tested negative for mycoplasma contamination.

Commonly misidentified lines
(See ICLAC register)

None of the used cell lines are listed in the ICLAC database.

Animals and other research organisms

Policy information about studies involving animals; ARRIVE guidelines recommended for reporting animal research, and Sex and Gender in Research

Laboratory animals

Nude female mice were purchased from Charles River, Crl:NMRI-Foxn1nu Strain Code 639 and used for in vivo studies at the age of 6-9 weeks. KB1P and KB2P mice strains were previously described (Evers et al. 2008; Bouwman et al. 2013; Duarte et al. 2017; Gogola et al. 2018). All mice were housed on standard 12 hour day/night cycle in ventilated cages with ad libitum food. Room temperature was maintained at 21 degrees Celsius and humidity was 55%.

Wild animals

No wild animals were used in this study.

Reporting on sex

All experiments were performed with female mice as this work focuses on breast cancer.

Field-collected samples

This study did not involve field sample collection.

Ethics oversight	All animal experiments were approved by the Animal Ethics Committee (BLV Bern, Switzerland, Application number BE40/18). All experiments were performed in accordance with the Swiss Act on Animal Experimentation (December 2015).

Note that full information on the approval of the study protocol must also be provided in the manuscript.

Plants

Seed stocks	No plant material was used in this study.
Novel plant genotypes	No plant material was used in this study.
Authentication	No plant material was used in this study.
